# Dietary fatty acid patterns and risk of metabolic syndrome: Tehran lipid and glucose study

**DOI:** 10.1186/s40001-023-01348-4

**Published:** 2023-09-20

**Authors:** Parvin Mirmiran, Zahra Gaeini, Zahra Feizy, Fereidoun Azizi

**Affiliations:** 1 Nutrition and Endocrine Research Center, Research Institute for Endocrine Sciences, Shahid Beheshti University of Medical Sciences, No. 24, Shahid-Erabi St., Yeman St., Velenjak, P.O. Box: 19395-4763, Tehran, Iran; 2 Department of Nutritional Sciences, Texas Tech University, Lubbock, TX 79409 USA; 3 Endocrine Research Center, Research Institute for Endocrine Sciences, Shahid Beheshti University of Medical Sciences, Tehran, Iran

**Keywords:** Fatty acid patterns, Metabolic syndrome, Saturated fats, Poly-unsaturated fats

## Abstract

**Background:**

The association between individual dietary fatty acids (FAs) and risk of metabolic syndrome (MetS) has been investigated in previous studies. However, synergistic or additive effects of multiple FA have received less attention. Hence, we aimed to determine the major dietary FA patterns and evaluate the association between FA patterns and risk of MetS.

**Methods:**

Dietary intakes of 1713 MetS-free adults who participated in the third phase of the Tehran Lipid and Glucose Study (TLGS) were assessed using a validated 168-items food frequency questionnaire. FA patterns were obtained by principal component analysis (PCA). Adjusted Hazard Ratios (HRs) and 95% confidence intervals (CIs) were calculated for the association of MetS incident with the extracted FA patterns.

**Results:**

Four major FA patterns were identified through PCA of the 24 FAs consumed: “short- and medium-chain saturated fatty acid (SFA) pattern”, “long-chain FA pattern”, “omega-3 PUFA pattern”, and “long-chain SFA pattern”. There was no significant association between dietary FA patterns and risk of MetS incidence.

**Conclusions:**

We found no significant association between FA patterns and risk of MetS. More prospective cohort studies and clinical trials are needed to clarify the issue.

## Introduction

Metabolic syndrome (MetS), which is defined as a clustering of abdominal obesity, hyperglycemia, hypertension, and atherogenic dyslipidemia, has serious implications on an individual’s health [1]. Among several known risk factors for the development of MetS, dietary intakes are important modifiable factors. The effect of total dietary fat and different types of fatty acids (FAs) on MetS and its components has previously been investigated, although the results are inconsistent. In a previous cohort study, a higher intake of vegetable fats was associated with a lower risk of developing MetS. In contrast, dietary intakes of total fat, animal fat, saturated fatty acid (SFA), and mono- and poly-unsaturated fatty acid (MUFA and PUFA) had no significant association with MetS [2]. On the other hand, a cross-sectional study reported that higher consumption of total fat and SFA was associated with a higher risk of MetS [3]. Among the individual components of MetS, previous studies reported inverse associations between vegetable fats [2], SFA, and omega-3 PUFA [4] and risk of hypertriglyceridemia. In addition, a higher intake of total fat, SFA, MUFA, and omega-3 PUFA was associated with a higher risk of hyperglycemia, and a higher intake of linoleic acid (LA) was associated with an increased risk of low HDL-c levels [4].

Although individual FAs can have differential effects on MetS, the combination of FAs may affect the risk of MetS in another way. The studies investigating the impact of individual types of FAs on metabolic health may have missed the synergistic or additive effects of intake of multiple fats. Analyzing the patterns of FAs, by shifting the focus from single FA to the pattern of all FAs, could dissolve these problems by uncovering the inter-relation of fats. To the best of our knowledge, there is very limited data regarding dietary FA patterns and their association with MetS. Noel et al. obtained four dietary FA patterns by principal components analysis (PCA), and suggested an inverse association between the “omega-3 PUFA/fish pattern” and MetS risk [5]. Another study reported an inverse association between the “long-chain SFA pattern” and risk of hyper-low density lipo-protein(LDL)–cholesterolemia and a positive association between “short- and medium-chain SFA pattern” and risk of hyper-LDL–cholesterolemia [6]. On the other hand, another study suggested a positive association between a pattern with low concentrations of LA and risk of MetS and an inverse association between a pattern with high loads of omega-3 PUFA and risk of MetS [7].

Considering the very limited and inconsistent data regarding dietary FA patterns and their association with metabolic abnormalities, this study aimed to characterize dietary FA patterns in Iranian adults and to examine the potential associations between FA patterns and risk of MetS.

## Methods

### Study population and measurements

The present cohort study was conducted within the framework of the Tehran Lipid and Glucose Study (TLGS). The first examination of TLGS initiated in 1999, and measurements were repeated every 3 years [8]. Adult participants of the third phase of the TLGS (2006–2008), who had a complete medical history and physical examination data, were recruited (*n* = 10091). After exclusion of the participants who had incomplete dietary data (*n* = 7036), participants with MetS at baseline (*n* = 959), participants who had incomplete data in terms of MetS (*n* = 130), participants with under- or over-reports of energy intakes (< 800 kcal/d or > 4200 kcal/d, respectively) (*n* = 106), and participants who lost to follow up (*n* = 147), 1713 MetS-free adults were remained and followed up to the sixth phase of the TLGS (2014–2017). It’s notable that there was no significant difference between the characteristics of participants who completed the FFQ at baseline and those of the total population in the third phase of TLGS [9]. The follow-up period’s median (inter-quartile range) was 7.6 (4.9–9.1) years from baseline.

### Anthropometric and demographic assessments

Anthropometric and demographic data were collected by trained interviewers of the TLGS. Body weight was measured to the nearest of 100 gr, using digital scales (Seca, Hamburg, Germany). Height was measured to the nearest of 0.5 cm in a standing position using a tape meter. Body mass index (BMI) was calculated as weight (kg) divided by the square of height (m^2^). Waist circumference (WC) was recorded to the nearest of 0.1 cm, using a soft measuring tape at the umbilicus, without any pressure on body surface. Anthropometric measurements were conducted, while the participants had minimal clothes, and no shoes.

Two measurements of blood pressure, with at least a 30 s interval, were taken to assess the systolic (SBP) and diastolic (DBP) blood pressures using a standard mercury sphygmomanometer calibrated by the Iranian Institute of Standards and Industrial Researches [10]. Before measuring SBP and DBP, participants remained seated for 15 min. The mean of the two measurements was considered as the final blood pressure.

To assess usual physical activity levels of participants, the Persian version of the modifiable activity questionnaire (MAQ), which was previously validated for participants of the TLGS [11], was used. Participants were asked to report the frequency and time spent on their activities of light, moderate, hard, and very hard intensity during the past 12 months, according to a list of common activities of daily life. Metabolic equivalent minutes per week (MET-min/week) was used to express physical activity levels [12]. Scores ≤ 600 METs-min/week were considered as low physical activity, and scores > 600 METs-min/week were considered as moderate and high physical activity.

### Biochemical measurements

Blood samples were taken after 12–14 h of overnight fasting, between 7:00 and 9:00 AM. Fasting serum glucose (FSG) and 2 h serum glucose (2 h-SG) levels were measured using an enzymatic colorimetric method with glucose oxidase. Serum triglyceride (TG) was assayed using an enzymatic colorimetric method, with glycerol phosphate oxidase. High-density lipoprotein-cholesterol (HDL-C) was measured after precipitation of the Apo-lipoprotein B containing lipoproteins with phosphotungstic acid. All blood analysis was done at the research laboratory of the TLGS, using Pars Azmoon kits (Pars Azmoon Inc., Tehran, Iran) and a Selectra 2 auto-analyzer (Vital Scientific, Spankeren, The Netherlands). Both inter- and intra-assay coefficients of variation (CV) at baseline and follow-up phase were less than 5%.

### Dietary assessment

We used a valid and reliable semi-quantitative 168-item food frequency questionnaire (FFQ) to assess the usual dietary intakes of participants at baseline [13]. Participants were asked to report their intake frequency for each food item consumed during the past year on a daily, weekly, or monthly basis. The frequencies were then converted to daily intakes, and portion sizes were reported in household measures and converted to grams [9]. We estimated the participant’s daily intakes of FAs by the USDA food composition table data. The 24 FAs used to determine FA patterns composed of 11 SFAs (including butyric acid (C4:0), caproic acid (C6:0), caprylic acid (C8:0), capric acid (C10:0), lauric acid (C12:0), myristic acid (C14:0), palmitic acid (C16:0), stearic acid (C18:0), arachidic acid (C20:0), behenic acid (C22:0) and lignoceric acid (C24:0)), 6 MUFAs (including myristoleic acid (C14:1), palmitoleic acid (C16:1), oleic acid (C18:1), gadoleic acid (C20:1), erucic acid (C22:1) and nervonic acid (C24:1)) and 7 PUFAs (including linoleic acid (C18:2, n-6), α-linolenic acid (C18:3, n-3), γ-linoleic acid (C18:3, n-6), eicosadienoic acid (C20:2, n-6), arachidonic acid (C20:4, n-6), eicosapentaenoic acid (C20:5, n-3), docosahexaenoic acid (C22:6, n-3)).

### Definition of terms and outcomes

We used the NCEP ATP III diagnostic criteria to define MetS [14]. Participants who had at least 3 of the following metabolic abnormalities were considered as MetS subjects: 1-hyperglycemia (FSG ≥ 100 mg/dL (5.6 mmol/L) or self-reported taking blood glucose-lowering medication); 2-Hypertriglyceridemia (serum TG ≥ 150 mg/dL (1.69 mmol/L) or using lipid-lowering drugs); 3-Low HDL-c (serum HDL-c < 40 mg/dL (1.04 mmol/L) for men and < 50 mg/dL (1.29 mmol/L) for women, or drug treatment); 4-hypertension (SBP/DBP ≥ 130/85 mm Hg or drug treatment for hypertension), and 5-Abdominal obesity (WC ≥ 95 cm for both genders). For WC, we used the modified cutoff points for Iranian adults [15].

### Statistical analyses

The PCA was used to determine patterns of dietary FAs, based on the 24 FAs, with varimax rotation and correlation matrix at baseline. To determine the number of factors to retain, we considered eigenvalues > 1, the scree plot, and the interpretability of the patterns. All 24 FAs contributed to the pattern score calculation; however, FAs with an absolute component loading score of ≥ 0.50 and < − 0.50 were selected to describe the patterns. The Kaiser–Meyer–Olkin statistic, a measure of sampling adequacy, was 0.49, and the *P* value for Bartlett’s test of sphericity was < 0.001. The factor scores for each extracted pattern were calculated using the sum of the frequency of consumption multiplied by factor loadings on each FA pattern. We identified four patterns and categorized factor scores into tertiles.

Baseline characteristics of participants were reported as mean (± SD) values for continuous variables and frequencies (%) for categorical variables and compared according to the tertiles of FA patterns scores, using ANCOVA. The incidence of MetS over the follow-up period was considered as a dichotomous variable (yes/no) in the models.

Cox proportional hazards regression models with person-years as the underlying time metric were used to estimate hazard ratios (HRs) and 95% confidence intervals (CIs) for the association between FA pattern and MetS incidence. Time to event for MetS was defined as time to end of follow-up (censored cases) or time to having an event, whichever occurred first. The proportional hazard assumption of the multivariable Cox model was assessed using Schoenfeld’s global test of residuals.

The univariate analysis was performed to obtain the final multivariable models and determine confounding variables. Variables with P_E_ less than 0.2 in the univariate analyses were selected as potential confounders. Confounders adjusted in the Cox models, included sex (men/women), age (years), BMI (kg/m^2^), smoking (yes/no), physical activity level (low/high), total energy (kcal/d), total protein (g/d) and total fiber (g/d) intake.

All statistical analyses were performed using the Statistical Package for Social Science (version 20; IBM Corp., Armonk, NY, USA), *P* values < 0.05 being considered significant.

## Results

The mean age (± SD) of the participants was 36.99 (± 13.12) years, and 40.9% of participants were men. The median (inter-quartile range) of follow-up duration was 7.6 (4.9–9.1) years and the incidence rate of MetS during that time was 34.8%.

Table 1 shows the absolute component loading scores of 24 main FAs, extracted by the use of PCA. However, all 24 FAs contributed to the pattern score calculation; FAs with an absolute component loading score of ≥ 0.50 and < − 0.50 were selected to describe the patterns. Four major FA patterns were identified, which explained 66.85% of the total variation of 24 main FAs. The first pattern was characterized by high loads of butyric acid, caproic acid, caprylic acid, capric acid, lauric acid, myristic acid, palmitic acid, stearic acid, arachidic acid, palmitoleic acid, and oleic acid. The second pattern had high positive correlation with arachidic acid, behenic acid, lignoceric acid, nervonic acid, linoleic acid, α-linolenic acid, γ-linoleic acid and eicosadienoic acid. Pattern 3 was characterized by high loads of gadoleic acid, arachidonic acid eicosapentaenoic acid, and docosahexaenoic acid. Behenic acid and lignoceric acid were highly loaded in the fourth pattern. The patterns were defined as “short- and medium-chain SFAs pattern”, “long-chain FAs pattern”, “omega-3 PUFAs pattern”, and “long-chain SFAs pattern”, respectively.

**Table 1:** Factor loading matrix and explained variances for major fatty acid patterns identified by factor analysis^1^

Fatty acids	Patterns			
	1	2	3	4
	Short- and medium-chain SFA pattern	Long-chain FAs pattern	Omega-3 PUFA pattern	Long-chain SFA pattern
Butyric acid (C4:0)	**0.846**	− 0.346		
Caproic acid (C6:0)	**0.860**	− 0.372		
Caprylic acid (C8:0)	**0.846**	− 0.408		
Capric acid (C10:0)	**0.800**	− 0.391		
Lauric acid (C12:0)	**0.587**	− 0.324		
Myristic acid (C14:0)	**0.840**	− 0.382		
Palmitic acid (C16:0)	**0.651**			
Stearic acid (C18:0)	**0.736**			0.496
Arachidic acid (C20:0)	**0.505**	**0.560**		− 0.460
Behenic acid (C22:0)		**0.627**		**0.654**
Lignoceric acid (C24:0)		**0.586**		**0.683**
Myristoleic acid (C14:1)				
Palmitoleic acid (C16:1)	**0.701**		0.349	
Oleic acid (C18:1)	**0.655**	0.314		
Gadoleic acid (C20:1)			**0.853**	
Erucic acid (C22:1)		0.459		
Nervonic acid (C24:1)		**0.544**		
Linoleic acid (C18:2, n-6)		**0.685**		− 0.468
α-linoleic acid (C18:3, n-3)	0.328	**0.757**		
γ-linoleic acid (C18:3, n-6)		**0.770**		
Eicosadienoic acid (C20:2, n-6)		**0.616**		
Arachidonic acid (C20:4, n-6)			**0.855**	
Eicosapentaenoic acid (C20:5, n-3)			**0.584**	
Docosahexaenoic acid (C22:6, n-3)			**0.858**	
Explained variance (%)	27.38	18.99	12.50	7.98
Cumulative explained variance (%)	27.38	46.38	58.88	66.85

^1^Principle Component Analysis (PCA) performed on 24 fatty acids. Fatty acids with loadings ≥ 0.50 and less than − 0.50 (in bold) are being characteristic for the four patterns; loadings less than 0.3 are suppressed

General characteristics of participants based on tertiles of FA patterns are shown in Table 2. There was no significant difference in general characteristics of participants between tertile categories of the “long-chain FAs pattern” and the “long-chain SFAs pattern”. The mean levels of serum HDL-C increased significantly across tertile categories of the “short- and medium-chain SFAs pattern” (*P* value < 0.05). The mean age of the participants increased significantly across tertile categories of the “omega-3 PUFA pattern” (*P* value < 0.05). Dietary intakes of participants across tertile categories of FA patterns are presented in Table 3. Total energy intake of participants decreased across tertile categories of the “long-chain SFAs pattern” (*P* value < 0.05). However, there was no significant difference in total energy intake across tertiles of other FA patterns. Participants in the highest tertile of the “short- and medium-chain SFAs pattern”, had a lower intake of total protein and a higher intake of SFA compared to the participants in the lowest tertile (*P* value < 0.05). Across tertile categories of the “long-chain FAs pattern”, dietary intakes of total protein, total fat, MUFA, and PUFA increased, and dietary intake of carbohydrates decreased (*P* value < 0.05). With increasing tertiles of the “omega-3 PUFAs pattern”, carbohydrate and total protein intakes increased; however, dietary intake of total fat, SFA, MUFA, and PUFA decreased (*P* value < 0.05). Finally, dietary intakes of carbohydrate and total protein decreased across tertile categories of the “long-chain SFAs pattern”, while dietary intakes of total fat, MUFA, and PUFA increased (*P* value < 0.05).

**Table 2:** Baseline characteristics of participants based on the fatty acid patterns

Baseline characteristics	Short- and medium-chain SFA pattern		Long-chain FAs pattern		Omega-3 PUFA pattern		Long-chain SFA pattern	
	T1	T3	T1	T3	T1	T3	T1	T3
Age, year	37.02 ± 12.81	36.84 ± 13.61	36.67 ± 12.22	37.34 ± 13.96	35.67 ± 12.19	37.75 ± 13.97*	36.11 ± 13.13	37.89 ± 13.62
Male, %	37.7	41.7	41.7	38.5	42.2	39.9	44.1	37.7
BMI, kg/m^2^	25.87 ± 4.54	25.69 ± 4.59	25.82 ± 4.67	25.62 ± 4.54	25.70 ± 4.42	25.72 ± 4.67	25.56 ± 4.56	25.73 ± 4.48
WC, cm	85.48 ± 11.93	84.91 ± 12.83	85.54 ± 12.17	84.88 ± 12.81	84.77 ± 12.61	85.48 ± 12.62	85.05 ± 12.41	85.15 ± 12.18
Current smoker, %	6.1	8.9	8.5	7.8	8.0	7.5	8.1	7.3
Low physical activity*, %	37.5	39.4	36.3	41.2	39.9	37.3	36.3	39.1
SBP, mmHg	106.9 ± 12.22	107.5 ± 14.34	106.6 ± 13.30	107.7 ± 14.20	106.7 ± 13.82	107.6 ± 13.95	107.8 ± 13.20	107.6 ± 14.41
DBP, mmHg	70.65 ± 8.88	71.24 ± 9.93	70.72 ± 9.11	71.33 ± 9.25	70.63 ± 9.49	70.38 ± 9.29	70.89 ± 9.26	70.76 ± 9.69
Serum TG, mg/dL	110.6 ± 53.43	106.8 ± 48.47	111.9 ± 49.24	106.3 ± 50.98	106.4 ± 47.98	107.9 ± 53.88	107.0 ± 48.38	108.0 ± 52.01
Serum cholesterol, mg/dL	179.8 ± 36.52	178.3 ± 36.80	179.8 ± 36.82	178.5 ± 36.21	176.4 ± 36.13	179.4 ± 37.40	178.1 ± 37.46	179.8 ± 36.90
Serum HDL-C, mg/dL	44.72 ± 9.89	46.14 ± 9.98*	45.44 ± 10.00	45.41 ± 10.39	45.42 ± 10.07	45.36 ± 9.72	45.18 ± 10.25	45.67 ± 10.18
FSG, mg/dL	85.83 ± 10.72	85.65 ± 12.51	85.97 ± 11.59	85.67 ± 10.98	85.47 ± 11.37	85.76 ± 11.02	85.73 ± 11.82	85.96 ± 10.83

Data represented as mean ± SD and percent

*BMI* body mass index, *WC* waist circumference, *SBP* systolic blood pressure, *DBP* diastolic blood pressure, *TG* triglyceride, *HDL-C* high-density lipoprotein cholesterol, *FSG* fasting serum glucose

^*^P value < 0.05

**Table 3:** Dietary intakes of participants based on the fatty acid patterns

Dietary intakes	Short- and medium-chain SFA pattern		Long-chain FAs pattern		Omega-3 PUFA pattern		Long-chain SFA pattern	
	T1	T3	T1	T3	T1	T3	T1	T3
Total energy, kcal/d	2284 ± 700.4	2272 ± 730.6	2254 ± 691.0	2242 ± 742.6	2295 ± 704.9	2264 ± 703.4	2427 ± 735.5	2163 ± 730.9*
Carbohydrates, en%	57.17 ± 6.29	57.04 ± 8.57	57.82 ± 6.65	56.49 ± 7.98*	56.24 ± 6.63	57.90 ± 8.24*	57.41 ± 5.91	56.11 ± 8.65*
Total proteins, en%	13.79 ± 2.22	13.37 ± 2.44*	13.51 ± 2.02	13.57 ± 2.61*	13.48 ± 2.18	14.03 ± 2.76*	13.81 ± 2.35	13.17 ± 2.49*
Total fat, en%	31.64 ± 6.15	32.05 ± 8.37	31.23 ± 6.24	32.40 ± 8.10*	32.79 ± 6.82	30.56 ± 7.56*	31.31 ± 5.11	33.17 ± 8.93*
SFA, en%	10.90 ± 7.98	11.19 ± 4.69*	10.50 ± 3.33	11.08 ± 8.46	11.28 ± 8.28	10.41 ± 3.79*	11.04 ± 8.25	10.53 ± 3.06
MUFA, en%	11.02 ± 2.72	11.00 ± 3.13	10.79 ± 2.56	11.35 ± 3.29*	11.46 ± 2.81	10.51 ± 2.98*	10.76 ± 1.82	11.77 ± 3.80*
PUFA, en%	6.56 ± 2.22	6.50 ± 2.34	6.52 ± 2.02	6.84 ± 2.64*	6.79 ± 2.39	6.28 ± 2.26*	6.31 ± 1.44	7.38 ± 3.10*

Data represented as mean ± SD and percent

*SFA* saturated fatty acids, *MUFA* mono-unsaturated fatty acids, *PUFA* poly-unsaturated fatty acids, *en%* percentage from total energy

^*^P value < 0.05

HRs (95% CIs) of MetS in relation to dietary FA patterns scores are shown in Table 4. There was no significant association between FA patterns scores and incidence of MetS in the crude and adjusted models.

**Table 4:** Hazard ratios (95% confidence intervals) of MetS according to the fatty acid patterns

	Tertiles of fatty acid patterns			Patterns as continuous variable	*P* value
	1	2	3		
Short- and medium-chain SFA pattern					
*Participants/cases (n/n)*	*571/213*	*571/189*	*571/194*		
HR (95% CI) Crude Model	1.00	0.85 (0.70–1.04)	0.89 (0.73–1.09)	0.90 (0.79–1.03)	0.144
HR (95% CI) Model 1	1.00	0.87 (0.71–1.07)	0.85 (0.70–1.04)	0.91 (0.80–1.05)	0.191
HR (95% CI) Model 2	1.00	0.87 (0.71–1.06)	0.85 (0.70–1.03)	0.91 (0.79–1.04)	0.173
Long-chain FAs pattern					
*Participants/cases (n/n)*	*571/204*	*571/211*	*571/181*		
HR (95% CI) Crude Model	1.00	1.05 (0.86–1.27)	0.87 (0.71–1.07)	0.92 (0.81–1.05)	0.217
HR (95% CI) Model 1	1.00	1.04 (0.86–1.27)	0.85 (0.69–1.05)	0.92 (0.81–1.05)	0.244
HR (95% CI) Model 2	1.00	1.05 (0.86–1.24)	0.84 (0.68–1.05)	0.92 (0.80–1.05)	0.216
Omega-3 PUFA pattern					
*Participants/cases (n/n)*	*571/197*	*571/205*	*571/194*		
HR (95% CI) Crude Model	1.00	1.07 (0.88–1.31)	0.98 (0.80–1.20)	0.98 (0.89–1.09)	0.778
HR (95% CI) Model 1	1.00	1.01 (0.83–1.23)	0.94 (0.76–1.15)	0.97 (0.87–1.09)	0.616
HR (95% CI) Model 2	1.00	1.01 (0.83–1.24)	0.95 (0.77–1.16)	0.98 (0.87–1.11)	0.783
Long-chain SFA pattern					
*Participants/cases (n/n)*	*571/187*	*571/197*	*571/212*		
HR (95% CI) Crude Model	1.00	1.07 (0.88–1.30)	1.11 (0.91–1.36)	1.00 (0.87–1.14)	0.918
HR (95% CI) Model 1	1.00	1.09 (0.89–1.34)	1.12 (0.91–1.37)	1.01 (0.87–1.16)	0.936
HR (95% CI) Model 2	1.00	1.11 (0.90–1.36)	1.12 (0.92–1.38)	1.00 (0.86–1.15)	0.972

Data are hazard ratio (95% confidence interval); proportional hazard Cox regression was used

Model 1 was adjusted for sex (men/women), age (years), body mass index (kg/m^2^), smoking (yes/no), physical activity level

Model 2 was additionally adjusted for dietary intake of total energy (kcal/d), total protein (g/d), and total fiber (g/d)

*HR* Hazard Ratio, *CI* confidence interval

## Discussion

In the present prospective cohort study, we identified four major FA patterns from 24 dietary FAs consumed, including “short- and medium-chain SFAs pattern”, “long-chain FAs pattern”, “omega-3 PUFAs pattern”, and “long-chain SFAs pattern”. No significant association was found between FA patterns and risk of developing MetS.

To the best of our knowledge, only one study has examined the association between dietary FA patterns and risk of MetS and its components [5]. The study reported four major FA patterns, including “short- and medium-chain SFA/dairy pattern”, “(n-3) fatty acid/fish pattern”, “very long-chain SFA and PUFA/oils pattern”, and “MUFA/trans fats pattern”, which are nearly similar to the patterns obtained in the present study. They have shown a reverse association between “n-3 fatty acid/fish pattern” and risk of MetS; however, in line with our results, there was no significant association between other FA patterns and MetS. In addition, they have shown reverse associations between “SFA/dairy pattern” and risk of hyperglycemia and between “n-3 fatty acid/fish pattern” and risk of hypertension.

Dietary SFA has traditionally been recognized as a major etiological factor in the development of cardiovascular disease (CVD). Therefore, dietary guidelines generally recommended to limit SFA intake to less than 10% of total energy or replacing them with PUFA and carbohydrates [16]. The recommendation to limit SFA intake mostly comes from the well-demonstrated ability of SFA to raise the plasma LDL-C levels by reducing hepatic LDL-C receptor activity [17–19]. Despite this theory, the evidence from both cohort studies and randomized trials does not support the assertion that further restriction of dietary SFA will reduce clinical events [20, 21]. In addition, it has been previously shown that dietary SFA increases the level of large cholesterol-enriched LDL, which are less effective in developing increases is CVD than the small, dense LDL-C particles [22, 23]. On the other hand, SFA could increase HDL-C level and improve total cholesterol to HDL-C ratio, a robust marker of CVD risk [24]. In conclusion, considering the inconsistent results of the recent studies regarding the unfavorable effects of SFA intake on metabolic health, it seems that it is necessary to emphasize more on the quality of macronutrients instead of focusing on low SFA diets.

Studies have shown that different types of SFA, based on their dietary source and carbon chain length, act metabolically different and thus have different health effects. Several studies suggested that palmitic acid (C16:0), as a major dietary long-chain SFA in the Western dietary pattern, could increase the CVD risk by increasing total cholesterol and LDL-C levels [25, 26]. A recent review of observational studies and clinical trials suggested that consumption of dairy products, which are major dietary sources of long-chain SFAs, including palmitic acid (C16:0) (35% of total FAs), followed by stearic acid (C18:0) and myristic acid (C14:0), was associated with decreased risks of MetS, hyperglycemia and insulin resistance. Although, no conclusive result was found between dairy consumption and the risk of dyslipidemia [27]. Moreover, a randomized controlled trial showed that consumption of coconut oil, as a rich source of medium-chain SFAs, decreased TG levels [28]. In addition, previous observational studies and clinical trials had shown inconsistent results regarding the association between FA patterns and risk of hypertension. Supplementation with coconut oil did not affect blood pressure in two previous clinical trials among MetS and hypertensive subjects [28, 29], while higher intake of total SFAs was associated with an increased risk of hypertension among middle-aged and older women in a cohort study [30], and higher intake of total, medium- and long-chain SFAs were associated with a lower risk of hypertension among elderly subjects, in a cross-sectional study [31]. The differences in study designs (observational or experimental) or different amounts of daily SFA intake by participants may explain the inconsistencies in the results. Moreover, it has been shown that genetic predisposition could modulate the relationship between dietary SFA and metabolic abnormalities [32]. Taken together, these indicate the need for cohort studies with extended follow-up time to clarify the association between dietary short-, medium- and long-chain SFAs and risk of metabolic abnormalities.

In line with our findings regarding the null association between “omega-3 PUFAs pattern” and risk of MetS, two meta-analyses of case–control and cross-sectional studies reported no significant association between dietary intake of omega-3 PUFAs or fish and MetS risk [33, 34]. However, prospective cohort studies investigating the long-term association between omega-3 PUFA or fish intake and risk of MetS are limited and have conflicting results of null association [35, 36] or protective association [37–39].

Taken together, the results on the relation between dietary FA and risk of metabolic abnormalities are still inconsistent; recent data have provided new challenging evidence suggesting that the relationship between dietary fats and MetS may not be as straightforward as initially thought. Current evidence strongly supports the necessity of considering combination of FAs in diet instead of single FAs. In addition, adhering to a healthy dietary pattern emphasizing high-quality fats should be recommended.

Some limitations of the present study should be taken into account. First, we did not consider the changes in an individual’s diet and other MetS risk factors during the study follow-up, which may lead to some degree of misclassifications and biased estimated HRs. Second, the residual confounders from unknown factors that were not adjusted in the models should be considered. Third, as with any cohort study, we cannot report any causation between dietary FA patterns and risk of incident MetS. Finally, we had some degree of measurement errors due to the self-report questionnaires. The present study had some strong points, including the prospective design of the study, long follow-up period, using a validated FFQ, and representativeness of the general population. In addition, we used PCA to derive FA patterns using individual FA intakes to consider the combination of dietary FAs simultaneously, which was the positive point of our study.

## Conclusions

To conclude, we found no significant association between dietary FA patterns and risk of MetS incidence. More prospective cohort studies and clinical trials are needed to clarify the association between FA patterns and risk of MetS identifies the optimal combination of dietary FAs for metabolic health.

## Data Availability

The data sets used and/or analyzed during the current study available from the corresponding author on reasonable request.

## Abbreviations

BMI: Body mass index
CI: Confidence intervals
DBP: Diastolic blood pressure
FA: Fatty acid
FFQ: Food frequency questionnaire
HDL-C: High-density lipoprotein cholesterol
HR: Hazard ratios
LDL-C: Low-density lipoprotein cholesterol
MET: Metabolic equivalent
MetS: Metabolic syndrome
MUFA: Mono-unsaturated fatty acid
PUFA: Poly-unsaturated fatty acid
SFA: Saturated fatty acid
SBP: Systolic blood pressure
TLGS: Tehran lipid and glucose study
TG: Triglyceride
